# Efficacy of Repeated Botulinum Toxin Type A Injections for Spastic Equinus in Children with Cerebral Palsy—A Secondary Analysis of the Randomized Clinical Trial

**DOI:** 10.3390/toxins9080253

**Published:** 2017-08-21

**Authors:** Bo Young Hong, Hyun Jung Chang, Sang-Jee Lee, Soyoung Lee, Joo Hyun Park, Jeong-Yi Kwon

**Affiliations:** 1 Department of Rehabilitation Medicine, St. Vincent’s Hospital, College of Medicine, The Catholic University of Korea, Suwon 16247, South Korea; byhong@catholic.ac.kr; 2 Department of Physical Medicine & Rehabilitation, Samsung Changwon Hospital, Sungkyunkwan University School of Medicine, Changwon 51353, South Korea; reh.chj@gmail.com; 3 Department of Rehabilitation Medicine, Daejeon St. Mary’s Hospital, College of Medicine, The Catholic University of Korea, Daejeon 34943, South Korea; upperlimb@catholic.ac.kr; 4 Department of Physical Medicine & Rehabilitation, Keimyung University, Dongsan Medical Center, Daegu 41931, South Korea; sylee@dsmc.or.kr; 5 Department of Rehabilitation Medicine, Seoul St. Mary’s Hospital, College of Medicine, The Catholic University of Korea, Seoul 06591, South Korea; drpjh@catholic.ac.kr; 6 Department of Physical and Rehabilitation Medicine, Samsung Medical Center, Sungkyunkwan University School of Medicine, Seoul 06351, South Korea

**Keywords:** cerebral palsy, spasticity, botulinum toxin, onabotulinumtoxin A, letibotulinumtoxin A, equinus

## Abstract

Botulinum toxin A is considered an important tool to control spasticity in children with cerebral palsy. Several factors are known to affect the efficacy of botulinum toxin, such as dosage, appropriate muscle selection and application, age, and accompanying therapy. A multicenter, double-blind, randomized, prospective phase III clinical trial of botulinum toxin A for the treatment of dynamic equinus in 144 children with cerebral palsy was performed to compare the efficacies of letibotulinumtoxin A and onabotulinumtoxin A. Secondary analyses were performed to evaluate factors that affected the outcome, focusing on the number of times injections were repeated. Effectiveness was defined as a change of 2 or more in the physician’s rating scale. Multivariate regression analyses were performed with multiple variables. The first injection of botulinum toxin A significantly improved D subscale of Gross Motor Function Measure-88 scores at 3 months compared to repeated injections (*p* < 0.05). After 6 months, patients who had one injection or none before the study showed significantly better outcomes than those who had more than one injection in terms of observational gait scores.

## 1. Introduction

Cerebral palsy (CP), a group of disorders in movement and posture resulting from non-progressive injury of the immature brain [1], is the most common cause of disability in the pediatric population [2,3]. Among various subtypes grouped according to neurologic dysfunction (spastic, hypotonic, dystonic, athetotic, or others), the spastic type is the most common. It can involve unilateral or bilateral sides [4,5,6] and accounts for 70–80% of CP [5,7,8]. Spasticity is defined as a velocity-dependent increase in tonic stretch reflexes (muscle tone) with exaggerated tendon jerks, resulting from hyperexcitability of the stretch reflex, as one component of the upper motor neuron syndrome [9]. In CP, spasticity is known to increase up to 4 years of age, after which it decreases up to 12 years of age [10]. Spasticity results in the shortening of myotendinous units, joint contracture, bony deformity, joint subluxation or dislocation, abnormal posture, and pathologic gait [3]. Identifying the severity and distribution of spasticity is important in decisions about the options for managing spasticity [11]. Physiotherapy and occupational therapy remain the mainstays of treatment in spastic CP [3]; botulinum toxin A (BoNT) is widely used for spasticity control, and many studies support its safety and efficacy [12,13,14].

BoNT has been commonly used to reduce muscle spasticity in children with CP since the first report of its use in 1993 [15]. BoNT selectively and reversibly blocks the release of acetylcholine in the neuromuscular junction, thus reducing muscle overactivity [3,14,16]. Although there are general side effects, injections of BoNT are known to be relatively safe [17,18]. Oral medications have more systemic adverse effects, such as drowsiness and sedation [19], which make it hard to maintain the medications, especially in children. One of the most common indications for BoNT in ambulatory children with spastic CP involves the need to improve gait patterns [12,19]. Furthermore, BoNT also has positive effects on upper limb function and the kinematics of gait [19,20,21].

The response to the treatment depends on the dosage of BoNT, appropriate administration, and adjunctive rehabilitation therapies [20,22,23,24,25,26]. However, there is sometimes no effect after injection of BoNT. Secondary unresponsiveness has been thought to be related to antibody formation [27], but antibodies were infrequently detected in recent studies, including this clinical trial [26,28]. More common causes of unsatisfactory outcomes might be the wrong choice of or missed target muscles, inappropriate indications, or a lack of complementary rehabilitation treatment [12]. Several studies have been performed to identify factors that affect the efficacy of BoNT in the management of spasticity of the lower extremities. Fazzi et al. showed that, compared with those older than 48 months, children aged 48 months or younger showed significantly increased gross motor function measure (GMFM) scores after 3 months of injections. Additionally, those who could walk with support showed a significantly greater increase in GMFM scores than non-walkers [29]. Repeated injections of BoNT have long-term effects on gross motor function in children with CP, but the short-term effect (4 weeks) on functional improvement and reduction of spasticity was significantly higher after the first injection than after the last injection [30]. Kristina et al. reported that repeated injections produced an immediate reduction in muscle tone; however, the improvement in range of motion was brief, and was noted only after the first injections [31]. In a systematic review, Kahraman et al. concluded that the first two injections (one repeat) are especially effective for relieving spasticity and improving fine and gross motor activities [13].

A multicenter, double-blinded, randomized, prospective phase III clinical trial (*n* = 144, 92 boys) of BoNT for the treatment of dynamic equinus in children with CP was undertaken recently with the primary intention of comparing the efficacy of letibotulinumtoxin A (Botulax; Hugel, Chuncheon, South Korea) and onabotulinumtoxin A (Botox; Allergan, Irvine, CA, USA). The study found injection of letibotulinum toxin A to be as effective and safe as that of onabotulinum toxin A for the treatment of dynamic equinus foot deformity in children with spastic CP (co-submitted) [32]. Secondary analyses of data from those patients who completed the trial were performed to evaluate the factors that affect the efficacy of BoNT for dynamic equinus in children with CP. This report provides a more detailed evaluation in the context of these factors, focusing on the number of times the injections were repeated, which might have influenced the observational gait analysis scores and gross motor outcomes of BoNT.

## 2. Results

### 2.1. Physician’s Rating Scale

A physician’s rating scale for gait (PRS), based on that used in a trial of BoNT calf injections, was employed [33]. A PRS score difference of 2 or more was considered a successful response. The numbers and percentages of successful responses in terms of PRS scores at 6, 12, and 24 weeks after BoNT injection in the calf muscles are described in Table 1. There was a significant difference in efficacy at 6 weeks; children who had had two or fewer injections of BoNT before the study showed a better effect compared to those who had had more than two prior BoNT injections. However, there were no significant differences in the success rate between children who were first injected versus those who had been injected before, or between children who had received one or no injection versus those who were injected more than once, at 6, 12, and 24 weeks after BoNT injections. Additionally, there was no significant differences according to the limb involvement pattern (i.e., unilateral or bilateral). However, the proportion of children for whom the treatment was effective (PRS ≥ 2) was marginally greater, as shown by increases in the Gross Motor Function Classification System (GMFCS) level, after 24 weeks of injection (*p* = 0.05).

### 2.2. Gross Motor Function Measurement

Gross Motor Function Measure-88 (GMFM-88) total and subgroup D and E scores were evaluated to assess gross motor function. GMFM-88 scores are known to be useful outcome measures for detecting changes in gross motor function in children with CP undergoing interventions [31]. The standing dimension D includes 13 items that deal with various aspects of standing. The walking, running, and jumping dimension E includes 24 items that evaluate a variety of activities that begin with standing [33]. Subgroup D scores were significantly more improved in botulinum toxin-naïve patients compared to patients who had had previous injections at 12 weeks (Table 2, *p* = 0.042). However, there were no significant differences in total, D, and E GMFM-88 scores at 24 weeks after injection according to the number of the previous injections (Table 2, *p* > 0.05). The mean age (SD) of the groups according to previous injection was significantly different: no previous injection (none) 3.86 (1.93), previous injection (yes) 5.66 (2.02); one or none 4.11 (1.98), more than one 5.99 (1.93); two or less 4.36 (1.98), more than two 6.39 (1.87) (*p* < 0.001).

### 2.3. Multivariate Regression Analyses

When patients were grouped according to the number of previous injections (none or one, two or more), the baseline PRS score was a significant predictor of outcome (PRS ≥ 2) at 12 weeks after injection (odds ratio = 0.825, 95% confidence interval: 0.686–0.992, *p* = 0.0410; Table 3). At 24 weeks after injection, patients with two or more previous injections had significantly poorer outcomes than those with none or one previous injection (odds ratio = 0.412, 95% confidence interval: 0.179–0.948, *p* = 0.0371; Table 4). When previous injection history was grouped as yes vs. none in the multivariate regression analyses, age, sex, baseline total PRS and GMFM-88 score, GMFCS level, type of CP (unilateral vs. bilateral), and previous injection history did not significantly impact BoNT efficacy (PRS ≥ 2) at 12 and 24 weeks after injection (Tables S1 and S2). When previous injection history was grouped as three or more vs. two or less, none of the factors significantly affected BoNT efficacy at 12 and 24 weeks after injection (data not shown).

## 3. Discussion

The effect of BoNT on neuromuscular junction continues for 12 to 16 weeks [34]. Because of the temporary effect, the injection needs to be repeated in some patients. As cerebral palsy is a lifelong disease, it is important to know the long-term effect and efficacy of repeated injections. Multivariate analyses showed that initial observational gait score (PRS) could affect the efficacy (PRS ≥ 2) at 12 weeks after injection, and efficacy was significantly better at 24 weeks in children who had injections once or not at all before the study than in those who had injections more than once. This result is similar to those in a recent systematic review including 13 original articles (893 children), which concluded that the first two injections were especially effective. However, this study focused on spasticity control and improvement of gross motor activities and not on gait quality [13]. The effect on range of motion and reduction of spasticity occurs immediately after injection and is greater with the first injection [30,31], but functional improvement or maintenance of the effect could occur later in the course of the continuum of care. The difference in the scores on the standing dimension of the GMFM-88 at 3 months after the injection was significantly greater in patients who had not previously been injected compared to those who had previous injections. The ability to maintain various standing positions and to perform specific tasks from the standing position might have improved because the stability of the base of support increased significantly more through management of spasticity with BoNT in the first-injected children compared to those who had injections before. However, the total GMFM-88 and the walking, running, and jumping dimension scores did not significantly differ between first-injection and repeated-injection groups. Papavasiliou et al. reported that an important improvement occurred in the GMFM (eight-point difference) at 3 months after the first injection, but the improvement decreased with later injections in GMFCS level I–V children [35]. Injected muscles varied according to clinical needs (e.g., calf muscles, hip adductors, hamstrings) [35], but only calf muscles including the gastrcocnemius and soleus were targeted in this study. Differences in the muscle selected and gross motor levels of participants might have contributed to different outcomes regarding gross motor function after BoNT injection. Also, the age should be considered in interpreting this result. The children who had no previous BoNT injection were significantly younger (3.86 vs. 5.66 years old) than those who had had injection before.

Weakness, disuse, and chronic spasticity results in muscle shortening and contracture, and these might contribute to the increase of contractures and deformities in children with CP with ageing [19]. Younger age is known to be related to greater improvement in gross motor function [29,36,37,38]. Previous studies have treated age as a continuous variable or categorized it as 4 or 5 years old, younger, or older. However, in our study, multiple regression analyses showed that age was not a significant determinant of efficacy as evaluated by the PRS at 12 and 24 weeks after BoNT injection. The participants were relatively young, and the range of age was not wide. The oldest child was 10 years old, and the mean and median ages were around 5 years old, which might be the reason for the differences between our results and those of previous reports. In a study on the prognosis for gross motor function, the age at which children were expected to achieve 90% of their potential GMFM-66 score was younger than 5 years old: that is, 4.8 and 3.7 years old for those in GMFCS levels I and III, respectively [39]. Also, an increase in the gross motor function score flattens after achievement of functions, which means that GMFCS level and age could affect the results. There may also have been a ceiling effect on the GMFM test itself [40,41], and this may have contributed to difficulty in detecting subtle changes of gross motor function.

At 24 weeks, the efficacy of BoNT measured by the PRS increased marginally as the GMFCS level increased. Although other factors were not calibrated, this means that BoNT could be more beneficial for the maintenance of the effect on the gait pattern at 6 months after the injection as the GMFCS level increases. The result differed from that of previous studies by Linder et al. and Papavasiliou et al., which found evident improvement in children at GMFCS level III [38], and reported that children at GMFCS level IV gained significant enough gross motor function to change their GMFCS level [35]. However, the muscles injected and the outcomes addressed were different from those in our study. Indeed, PRS scores, such as those on the GMFM-88, might not always be linked to improvements in gross motor function. Although the GMFM-88 is a useful outcome measure with which to detect changes in gross motor function in children with CP [42,43], it focuses on function and does not reflect the quality of movement.

As the previous studies showed different effective dose in diplegic and hemiplegic patients, hemiplegic patients required higher doses [37]. This study used different doses for patients with unilateral and bilateral involvement, 3 IU/kg and 4 IU/kg, respectively, for each triceps surae. Therefore, there was no difference in their response according to the involvements of limbs, unilateral or bilateral. This also suggests that a higher dosage is needed for the children with unilateral involvement compared to bilateral involvement for a similar effect.

There are several limitations in this study. First, additional or supplemental therapies are known to be a significant influence on the outcome of BoNT injection [20,26], and the number of therapies or activities and the time spent performing them were not considered in this study. Also, participants were strongly advised not to change their usual therapies or activities during the clinical trial, but changing the frequency or intensity of therapies was not a cause for dropout from the study and was not investigated. Second, there are several types of BoNT available in South Korea; however, our data did not include information about what kind of BoNT the patients received before the study. Therefore, we could not investigate the cross-reactivity of BoNT preparations made by different manufacturers. Additionally, because of the 6-month observation duration, some data were missing due to the level of patients’ compliance. However, data in this study were from a multicenter, double-blind, randomized, prospective clinical trial, and the observation period for 144 spastic CP children was 6 months [32]. The study provides evidence about the factors affecting outcomes, observational gait scores, and gross motor function after BoNT intervention. In conclusion, at 3 months, the first injection of BoNT for the control of dynamic tip-toeing was associated with more improvement than repeated injection with regard to the standing ability of children with CP. At 6 months after the injection, patients who had had injections once or never before the study showed significantly better outcomes in terms of observational gait scores (PRS). Age was not a significant factor affecting the efficacy (PRS) of BoNT at 12 and 24 weeks after injection.

## 4. Materials and Methods

The study received permission from independent research ethics committees at each investigational site. Enrolled subjects were randomly assigned to receive letibotulinumtoxin A injection (study group, *n* = 70) or onabotulinumtoxin A injection (control group, *n* = 73) at a ratio of 1:1, and they then received the corresponding investigational products. The random assignment code was generated using a block randomization method. Those codes were sealed until the trial was completed, except in cases of serious adverse events. Participants were ambulatory children (*n* = 143, 91 boys) with spastic CP (GMFCS levels I–III) who were 2–10 years old and showed dynamic equinus deformity, tip-toe gait. The target muscles were the triceps surae, gastrocnemius, and soleus. In the case of bilateral involvement (*n* = 95, diplegia or tetraplegia), the investigational product was administered to both legs at a dose of 6 U/kg body weight (3 U/kg for each leg); in the case of unilateral involvement (*n* = 48, hemiplegia), it was administered to the spastic leg at a dose of 4 U/kg body weight.

For the secondary analyses of this phase III clinical trial, intention-to-treat (ITT) analyses were performed. Continuous variables were presented as means and standard deviations, and categorical variables were presented as frequencies and percentages. Chi-square tests were performed to analyze the efficacy of BoNT injection in terms of injection history, paralysis type, and GMFCS level at 6, 12, and 24 weeks after the injection. Analysis of linear-by-linear associations of the proportion of children in whom the treatment was effective (PRS ≥ 2), which was performed with SPSS software, version 17.0 (SPSS for Windows 12.0, Chicago, IL, USA), and SAS software version 9.4 (SAS Institute, Cary, NC, USA) allowed examination according to GMFCS level. Multivariate logistic regression analyses were performed to identify the factors that affect the outcome of BoNT on observational gait pattern (PRS) and motor function (GMFM-88). The following variables were treated as candidates for the purpose of determining associations with the level of responsiveness to BoNT: (1) age; (2) history of previous BoNT injections (yes vs. none; more than one vs. one or none; more than two vs. two or fewer); (3) baseline total PRS score; (4) baseline GMFM-88 total score; (5) GMFCS level; and (6) paralysis type of CP (i.e., unilateral or bilateral involvement).

## Figures and Tables

**Table 1:** Differences in physician’s rating scale (PRS) scores at 6 weeks, 12 weeks, and 24 weeks after botulinum toxin injection.

Subgroups		Baseline—6 Weeks			Baseline—12 Weeks			Baseline—24 Weeks		
		PRS < 2	PRS ≥ 2	*p*	PRS < 2	PRS ≥ 2	*p*	PRS < 2	PRS ≥ 2	*p*
		N (%)	N (%)		N (%)	N (%)		N (%)	N (%)	
Pre-injection History	None	8 (16.7)	40 (83.3)		11 (22.9)	37 (77.1)		20 (41.7)	28 (58.3)	
	Yes	29 (30.5)	66 (69.5)	0.074	25 (26.3)	70 (71.1)	0.658	47 (50)	47 (50)	0.347
	0−1	14 (19.7)	57 (80.3)		16 (22.5)	55 (77.5)		28 (39.4)	43 (60.6)	
	>1	23 (31.9)	49 (68.1)	0.095	20 (27.8)	52 (72.2)	0.470	39 (54.2)	32 (44.4)	0.110
	≤2	19 (20.2)	75 (79.8)		24 (25.5)	70 (74.5)		41 (43.6)	52 (55.3)	
	>2	18 (36.7)	31 (63.3)	0.032	12 (24.5)	37 (75.5)	0.892	26 (53.1)	23 (46.9)	0.457
Type	Unilateral	14 (29.2%)	34 (70.8%)		11 (22.9%)	37 (77.1%)		22 (46.8%)	25 (53.2%)	
	Bilateral	23 (24.2%)	72 (75.8%)	0.523	25 (26.3%)	70 (73.7%)	0.658	45 (47.4%)	50 (52.6%)	0.95
GMFCS level	I	22 (24.4%)	68 (75.6%)		20 (22.2%)	70 (77.8%)		35 (39.3%)	54 (60.7%)	
	II	9 (26.5%)	25 (73.5%)		10 (29.4%)	24 (70.6%)		20 (58.8%)	14 (41.2%)	
	III	6 (31.6%)	13 (68.4%)	0.809	6 (31.6%)	13 (68.4%)	0.562	12 (63.2%)	7 (36.8%)	0.05

GMFCS: Gross Motor Function Classification System.

**Table 2:** Differences in GMFM-88 scores between baseline and at 12 and 24 weeks after botulinum toxin injection according to number of previous injections.

Interval (Weeks)	Previous Injection		GMFM-Total Score			GMFM-D Score			GMFM-E Score		
		N	Mean	SD	*p*	Mean	SD	*p*	Mean	SD	*p*
12	None	48	2.51	3.455		2.21	4.021		3.08	3.769	
	Yes	95	1.69	3.246	0.164	0.86	2.838	0.042	2.75	4.654	0.665
	0–1	71	2.11	3.035		1.73	3.533		2.39	3.404	
	>1	72	1.82	3.610	0.612	0.90	3.086	0.137	3.32	5.126	0.206
	≤2	94	1.99	3.135		1.56	3.355		2.38	3.957	
	>2	49	1.91	3.704	0.896	0.84	3.262	0.216	3.78	4.976	0.070
24	None	47	3.26	3.941		2.49	4.133		4.91	5.77	
	Yes	94	2.566	4.159	0.344	1.38	3.386	0.092	3.77	5.411	0.247
	0–1	70	3.119	4.044		2.23	3.990		4.30	5.494	
	>1	71	2.480	4.132	0.356	1.28	3.296	0.126	4.00	5.619	0.749
	≤2	92	2.825	4.038		1.92	3.847		4.10	5.475	
	>2	49	2.745	4.218	0.913	1.43	3.342	0.448	4.24	5.714	0.881

GMFM: Gross Motor Function Measure, SD: Standard Deviation.

**Table 3:** Multivariate regression analyses of variables on outcome (PRS ≥ 2) at 12 weeks after botulinum toxin injection, according to the number of previous injections (none or one, two or more).

Variables	Coeff	SE	OR	95% CI (OR)		*p* ^1^
Age	−0.1084	0.1038	0.897	0.732	1.100	0.2960
Gender						
Male	−0.1033	0.3986	0.902	0.413	1.970	0.7955
Female			1.000			
PRS Score	−0.1924	0.0942	0.825	0.686	0.992	0.0410
GMFCS Level						
I			1.000			
II	−0.4175	0.5709	0.659	0.215	2.016	0.4645
III	0.3337	0.9564	1.396	0.214	9.100	0.7272
GMFM Score	0.0364	0.0246	1.037	0.988	1.088	0.1393
Previous injection						
>1	−0.7322	0.4405	0.481	0.203	1.140	0.0965
0–1			1.000			
Type						
Bilateral	−0.5490	0.4737	0.578	0.228	1.462	0.2465
Unilateral			1.000			

^1^ *p* Value calculated by logistic regression analysis, GMFCS: Gross Motor Function Classification System, GMFM: Gross Motor Function Measure, OR: Odds Ratio, CI: Confidence Interval.

**Table 4:** Multivariate regression analyses of effects of variables on outcomes (PRS ≥ 2) at 24 weeks after botulinum toxin injection according to number of previous injections: more than one vs. one or none.

Variables	Coeff	SE	OR	95% CI (OR)		*p* ^1^
Age	0.1423	0.1040	1.153	0.940	1.414	0.1711
Gender						
Male	0.1184	0.3771	1.126	0.538	2.358	0.7535
Female			1.000			
PRS Score	−0.1747	0.0926	0.840	0.700	1.007	0.0592
GMFCS Level						
I			1.000			
II	−0.8834	0.5684	0.413	0.136	1.259	0.1201
III	−1.7753	0.9737	0.169	0.025	1.143	0.0683
GMFM Score	0.0005	0.0240	1.000	0.954	1.049	0.9850
Previous injection						
>1	−0.8871	0.4256	0.412	0.179	0.948	0.0371
0–1			1.000			
Type						
Bilateral	0.1873	0.4298	1.206	0.519	2.800	0.6630
Unilateral			1.000			

^1^ *p* Value calculated by logistic regression analysis, PRS: Physician’s Rating Scale, GMFCS: Gross Motor Function Classification System., OR: Odds Ratio, CI: Confidence Interval.

## Supplementary Materials

The following are available online at www.mdpi.com/2072-6651/9/8/253/s1, Table S1: Multivariate regression analyses of variables on outcome (PRS ≥ 2) at 12 weeks for botulinum toxin injection, previous injection groups: yes vs. none, Table S2: Multivariate regression analyses of variables on outcome (PRS ≥ 2) at 24 weeks for botulinum toxin injection, previous injection groups: yes vs. none.
