# Supplementary material for: Efficacy of Repeated Botulinum Toxin Type A Injections for Spastic Equinus in Children with Cerebral Palsy—A Secondary Analysis of the Randomized Clinical Trial
Source: Toxins (Basel). 2017 Aug 21;9(8):253. doi: 10.3390/toxins9080253 (PMC5577587; doi:10.3390/toxins9080253)
Supplement: Supplementary file 1 [file toxins-09-00253-s001.pdf]

Supplementary Materials: Efficacy of Repeated Botulinum Toxin Type A Injections for Spastic Equinus in Children with Cerebral Palsy—A Secondary Analysis of the Randomized Clinical Trial

Bo Young Hong, Hyun Jung Chang, Sang-Jee Lee, Soyoung Lee, Joo Hyun Park and Jeong-Yi Kwon

Table S1. Multivariate regression analyses of variables on outcome (PRS ≥ 2) at 12weeks for botulinum toxin injection, previous injection groups: yes vs. none.

Variables	Coeff	SE	OR	95% CI (OR)		*P*[1]
Age	0.2057	0.1301	1.228	0.952	1.585	0.1140
Gender						
Male	-0.2477	0.4518	0.781	0.322	1.892	0.5835
Female			1.000			
PRS Score	-0.1762	0.1119	0.838	0.673	1.044	0.1153
GMFCS Level						
I			1.000			
II	-0.2457	0.6282	0.782	0.228	2.679	0.6957
II	-0.5362	0.9856	0.585	0.085	4.037	0.5864
GMFM Score	0.00846	0.0261	1.008	0.958	1.061	0.7457
Previous injection						
Yes	-0.5729	0.5291	0.564	0.200	1.590	0.2788
None			1.000			
Type						
Bilateral	-0.5539	0.7500	0.575	0.132	2.500	0.4602
Unilateral			1.000			

[1] *P* value calculated by logistic regression analysis, GMFCS: Gross Motor Function Classification System, GMFM: Gross Motor Function Measure, OR: Odds Ratio, CI: Confidence Interval.

Table S2. Multivariate regression analyses of variables on outcome (PRS ≥ 2) at 24 weeks for botulinum toxin injection, previous injection groups: yes vs. none.

Variables	Coeff	SE	OR	95% CI (OR)		*P*[1]
Age	0.0372	0.0992	1.038	0.854	1.261	0.7080
Gender						
Male	-0.0601	0.3773	0.942	0.450	1.973	0.8735
Female			1.000			
PRS Score	-0.1163	0.0901	0.890	0.746	1.062	0.1968
GMFCS Level						
I			1.000			
II	-0.6027	0.5538	0.547	0.185	1.620	0.2764
II	-1.8946	1.0280	0.150	0.020	1.128	0.0653
GMFM Score	0.000164	0.0245	1.000	0.953	1.049	0.9947
Previous injection						
Yes	-0.3234	0.4302	0.724	0.311	1.682	0.4523
None			1.000			
Type						
Bilateral	-0.2773	0.4152	0.758	0.336	1.710	0.5042
Unilateral			1.000			

[1] *P* value calculated by logistic regression analysis, GMFCS: Gross Motor Function Classification System, GMFM: Gross Motor Function Measure, OR: Odds Ratio, CI: Confidence Interval.
